# “Shut down and restart”: immersion in rebirth micro-dramas and narrative career disruption among Chinese university students—an interpretative phenomenological analysis

**DOI:** 10.3389/fpsyg.2026.1887188

**Published:** 2026-07-22

**Authors:** Peitao Du, Tingting Liu, Xujin Xian

**Affiliations:** 1 Al-Farabi Kazakh National University, Almaty City, Kazakhstan; 2 Department of Education, The Catholic University of Korea, Bucheon City, Republic of Korea

**Keywords:** career construction, Chinese university students, digital media, interpretative phenomenological analysis, meaning in life, psychological capital, rebirth micro-drama

## Abstract

Rebirth micro-dramas have become a popular form of short-form entertainment in China. In these stories, a protagonist dies or is betrayed, returns to an earlier life stage with memories of the future, avoids previous mistakes, and quickly achieves success. For university students approaching graduation, this narrative may be especially attractive because it offers what real career development cannot: certainty, reversibility, and foreknowledge. Real career choices are slow, uncertain, and irreversible, and students must continue investing effort without knowing whether their decisions will lead to success. This study used interpretative phenomenological analysis to examine how 18 graduating undergraduate and final-year master's students in China understood rebirth or comeback-themed micro-dramas in relation to their own career difficulties and sense of meaning in life. All participants reported frequent exposure to such dramas and current career-related distress. Semi-structured interviews were conducted. The Self-Rating Idea of Suicide Scale, the Career Decision-Making Difficulties Questionnaire, and the Meaning in Life Questionnaire were used only for purposive screening, case-level contextualization, and interpretative triangulation, not for diagnosis, statistical comparison, hypothesis testing, or causal inference. Three superordinate themes were identified. First, participants described a disenchanted comeback script, in which fictional certainty and rapid success made their own career development feel delayed, uncertain, and wrongly plotted. Second, some participants reported a perceived erosion of resilience, because ordinary effort seemed weak when compared with fictional success based on foreknowledge, hidden advantage, or instant reversal. Third, for several participants in higher-risk contextual profiles, reboot fantasies became entangled with meaning loss, emotional escape, and death-related metaphors. These accounts are interpreted as expressions of existential distress rather than evidence that micro-drama viewing directly produced clinical risk. Based on these findings, we propose narrative career disruption to describe how rebirth narratives may complicate vulnerable students‘ attempts to build realistic, agentic, and temporally continuous career stories. The study contributes to vocational psychology, cyberpsychology, and positive organizational behavior by showing how digital success narratives may shape young adults' interpretations of effort, failure, resilience, and life meaning during the school-to-work transition.

## 1 Introduction

What happens when the fantasy of restarting life becomes a routine media script through which young people interpret failure, uncertainty, and future possibility? In contemporary China, short-form video and micro-drama platforms have become a highly visible part of young adults' everyday media consumption. Among various genres, rebirth micro-dramas have gained particular popularity. These dramas usually follow a stable narrative formula: the protagonist dies or is betrayed, returns to a decisive earlier moment in life, retains memories from the previous timeline, uses this informational advantage to avoid mistakes, and achieves rapid revenge, wealth, social recognition, or romantic success. Episodes are short, emotionally intense, and structured around fast rewards (Montag et al., 2021; Zhang et al., 2019; Qin et al., 2022; Liu et al., 2021; Chen et al., 2023). For university students facing academic competition, employment pressure, social comparison, and uncertainty about future identity, such narratives may offer temporary relief from the slow and uncertain process of real-life development (Xie et al., 2023; Mao and Liao, 2025; Verduyn et al., 2020; Valkenburg et al., 2022; Odgers and Jensen, 2020; Coyne et al., 2020; Green and Brock, 2000; Moyer-Gusé, 2008; Cohen, 2001).

Existing research on short-form video use has mainly focused on addiction, perceived stress, attention, academic procrastination, learning burnout, and social comparison (Montag et al., 2021; Zhang et al., 2019; Qin et al., 2022; Liu et al., 2021; Chen et al., 2023; Xie et al., 2023; Mao and Liao, 2025; Verduyn et al., 2020). Broader studies of digital media and youth mental health have also emphasized that media effects should not be reduced to screen time alone, but should be understood in relation to content, context, and individual vulnerability (Valkenburg et al., 2022; Odgers and Jensen, 2020; Coyne et al., 2020). These perspectives are important, yet they leave insufficiently examined how specific digital narrative structures shape young adults' self-understanding. Rebirth micro-dramas are theoretically relevant because they do not merely provide entertainment; they offer a compressed success script in which uncertainty is removed, mistakes can be reversed, and success depends less on gradual effort than on privileged foreknowledge. Research on narrative transportation, entertainment persuasion, and identification suggests that fictional narratives may influence viewers' beliefs, emotions, and self-relevant interpretations when they become absorbed in story worlds or identify with media characters (Green and Brock, 2000; Moyer-Gusé, 2008; Cohen, 2001).

The school-to-work transition provides a particularly important context for examining this issue. Career construction theory views career development as a process through which individuals connect past experiences, present choices, and future aspirations into a meaningful life-career narrative (Wang and Li, 2024; Savickas and Porfeli, 2012; Rudolph et al., 2017; Savickas et al., 2009). From this perspective, career difficulties are not only problems of information, matching, or decision-making; they may also involve disruptions in the stories through which young adults understand effort, failure, time, and agency (Pordelan et al., 2021, 2018; Gati et al., 1996; Kulcsár et al., 2020; Xu and Tracey, 2015). Psychological capital theory provides a complementary lens by emphasizing self-efficacy, hope, optimism, and resilience as psychological resources that support persistence and adaptive coping (Luthans and Youssef-Morgan, 2017; Avey et al., 2011; Newman et al., 2014; Luthans et al., 2010). However, these resources are embedded in cultural and digital environments. Repeated exposure to narratives in which success is immediate, certain, and dependent on a “restart” may influence how vulnerable viewers interpret ordinary setbacks and long-term effort.

Although rebirth-themed short dramas are widely consumed, their psychological meaning remains underexplored. Existing studies have provided valuable evidence on short-form video use and youth well-being, but they have paid less attention to how a specific narrative formula may interact with career distress, weakened meaning in life, or suicidal ideation (Steger et al., 2006; Martela and Steger, 2016; George and Park, 2016; Heintzelman and King, 2014; Arnett, 2000; Van Orden et al., 2010; Franklin et al., 2017; Xia et al., 2002). The central issue is not whether rebirth micro-dramas mechanically cause psychological distress. Rather, the question is how certain viewers interpret these narratives in relation to their own developmental struggles. For some students, rebirth stories may remain harmless entertainment. For others, especially those already experiencing career indecision, hopelessness, or a reduced sense of meaning, the fantasy of restarting life may become a symbolic resource for interpreting failure, withdrawal, or escape from an irreversible life course. This content-specific and vulnerability-sensitive perspective helps avoid reducing digital media effects either to screen time alone or to a deterministic claim about narrative harm.

To address this gap, the present study explored the lived experiences of graduating undergraduate and final-year master's students in China who frequently watched rebirth or comeback-themed micro-dramas and reported current career-related distress. Drawing on career construction theory and psychological capital theory, this study used interpretative phenomenological analysis to examine how participants made sense of career success, failure, persistence, withdrawal, and the fantasy of rebooting life (Smith, 1996; Pietkiewicz and Smith, 2014). The study therefore treats rebirth micro-dramas as interpretative resources within participants' meaning-making, rather than as isolated media stimuli. The study was guided by three research questions: RQ1: How do graduation-stage Chinese university students who frequently watch rebirth micro-dramas make sense of career success, failure, and uncertainty? RQ2: How do participants interpret the relationship between rebirth narratives and their own persistence, withdrawal, or career decision-making difficulties? RQ3: In what ways does the fantasy of rebooting life become connected with meaning in life, avoidance, or suicidal ideation among students experiencing career-related distress? (Wang and Li, 2024; Savickas and Porfeli, 2012; Rudolph et al., 2017; Savickas et al., 2009; Pordelan et al., 2021, 2018; Gati et al., 1996; Kulcsár et al., 2020; Xu and Tracey, 2015; Luthans and Youssef-Morgan, 2017; Avey et al., 2011; Newman et al., 2014; Luthans et al., 2010; Steger et al., 2006; Martela and Steger, 2016; George and Park, 2016; Heintzelman and King, 2014; Arnett, 2000; Van Orden et al., 2010; Franklin et al., 2017; Xia et al., 2002).

The following sections review the relevant literature and theoretical framework, describe the interpretative phenomenological research design, present the qualitative findings, and discuss the implications for vocational psychology, cyberpsychology, positive organizational behavior, university counseling, and media literacy.

## 2 Literature review and theoretical framework

### 2.1 Rebirth micro-dramas as digital success scripts

Rebirth micro-dramas are a distinctive genre of short-form serial storytelling. Their typical structure involves death, temporal return, privileged knowledge, accelerated revenge, and ultimate success. Unlike traditional coming-of-age stories that emphasize gradual development, moral ambiguity, and learning through error, rebirth narratives often eliminate uncertainty. The protagonist succeeds because they already know what will happen. In narrative terms, the future is not discovered; it is exploited (Montag et al., 2021; Zhang et al., 2019; Qin et al., 2022; Liu et al., 2021; Chen et al., 2023; Green and Brock, 2000; Moyer-Gusé, 2008; Cohen, 2001).

The medium-specific features of rebirth micro-dramas also distinguish them from legacy temporal fantasies such as long-form time-loop films, reincarnation novels, or Japanese isekai-style stories. The rebirth motif itself is not new; what is distinctive in the contemporary micro-drama ecology is the compression of exposition, conflict, reversal, and reward into brief serial episodes, the high frequency of cliff-hangers, and the algorithmic delivery of adjacent plots with similar emotional payoffs. This structure can intensify narrative transportation because viewers do not need to wait for extended world-building or slow character development; instead, each episode quickly renews the contrast between humiliation and reversal, loss and comeback, uncertainty and foreknowledge. In this sense, rebirth micro-dramas combine the symbolic appeal of counterfactual fiction with the rapid reward cadence of short-form platforms (Montag et al., 2021; Zhang et al., 2019; Qin et al., 2022; Liu et al., 2021; Chen et al., 2023; Green and Brock, 2000; Moyer-Gusé, 2008; Cohen, 2001).

For viewers, such a narrative may function as a fantasy of restored agency. It offers the imagined possibility of correcting earlier mistakes, choosing the right major, avoiding the wrong relationship, investing in the right opportunity, or defeating those who previously caused humiliation. In contexts where young adults feel powerless, such stories may provide psychological compensation. However, the same structure may also sharpen the contrast between fictional certainty and real-world ambiguity (Green and Brock, 2000; Moyer-Gusé, 2008; Cohen, 2001; Gati et al., 1996; Kulcsár et al., 2020; Xu and Tracey, 2015). The present study therefore approaches rebirth micro-dramas not as uniform risk content, but as digital success scripts whose meaning depends on viewers' developmental position, emotional vulnerability, and interpretative distance from fiction.

### 2.2 Career construction theory and narrative career meaning

Career construction theory conceptualizes career development as a process through which individuals impose meaning on vocational behavior and construct a coherent life story (Wang and Li, 2024; Savickas et al., 2009). In contemporary societies, careers are less often defined by stable organizational paths and more often by subjective adaptation to changing opportunities, constraints, and identities. Career adaptability—commonly described through concern, control, curiosity, and confidence—supports individuals in preparing for the future, making choices, exploring possibilities, and trusting their capacity to act (Savickas and Porfeli, 2012; Rudolph et al., 2017).

From this perspective, career difficulties are not only problems of information or matching. They may also involve narrative disruption: the inability to connect past experiences, present choices, and future aspirations into a story that feels livable and meaningful (Wang and Li, 2024; Savickas et al., 2009; Pordelan et al., 2021, 2018). Rebirth micro-dramas are theoretically relevant because they offer an alternative to career construction. Rather than cultivating curiosity in the face of uncertainty, they imagine a world in which uncertainty has already been removed (Green and Brock, 2000; Moyer-Gusé, 2008; Cohen, 2001; Gati et al., 1996; Kulcsár et al., 2020; Xu and Tracey, 2015).

### 2.3 Psychological capital and the meaning of effort

Psychological capital refers to a positive psychological state characterized by self-efficacy, hope, optimism, and resilience (Luthans and Youssef-Morgan, 2017; Avey et al., 2011; Newman et al., 2014; Luthans et al., 2010). It has been widely used in organizational behavior and applied psychology to explain performance, well-being, employability, and adjustment. Although it originated in work and organizational contexts, its core constructs are relevant to students' academic and career development (Luthans and Youssef-Morgan, 2017; Avey et al., 2011; Newman et al., 2014; Luthans et al., 2010).

The present study extends psychological capital theory by examining participants' accounts of a possible narrative pathway through which psychological resources may be experienced as weakened. Traditional accounts often focus on how repeated failure weakens self-efficacy or resilience (Luthans and Youssef-Morgan, 2017; Avey et al., 2011; Newman et al., 2014; Luthans et al., 2010). The present study does not assume that digital media directly erodes these resources. Rather, it examines how some vulnerable viewers interpreted repeated exposure to narratives in which success depends on foreknowledge, hidden advantage, or magical reversal, and how these interpretations made ordinary effort feel less meaningful before sustained effort had fully begun (Green and Brock, 2000; Moyer-Gusé, 2008; Cohen, 2001).

### 2.4 Meaning in life, existential distress, and reboot fantasy

Meaning in life involves both the presence of meaning and the search for meaning (Steger et al., 2006; Martela and Steger, 2016; George and Park, 2016; Heintzelman and King, 2014). A temporary search for meaning may be developmentally normal, especially during emerging adulthood (Arnett, 2000). However, when high searching is combined with a low sense of meaning, individuals may experience distress, emptiness, or identity instability (Steger et al., 2006; Martela and Steger, 2016; George and Park, 2016; Heintzelman and King, 2014). Career uncertainty can intensify this condition because work and future planning are central to young adults' sense of direction (Wang and Li, 2024; Savickas and Porfeli, 2012; Rudolph et al., 2017; Savickas et al., 2009; Pordelan et al., 2021, 2018; Gati et al., 1996; Kulcsár et al., 2020; Xu and Tracey, 2015).

The rebirth narrative has existential significance because it symbolically reverses the irreversibility of life. In ordinary life, choices are consequential because time cannot be restarted. In rebirth micro-dramas, however, death becomes a gateway to correction. For most viewers, this is clearly recognized as fiction. For vulnerable viewers, however, the metaphor of rebooting may become emotionally charged (Green and Brock, 2000; Moyer-Gusé, 2008; Cohen, 2001; Van Orden et al., 2010; Franklin et al., 2017; Xia et al., 2002). For this reason, the present study examines reboot-related language as part of participants' meaning-making and risk context, while avoiding any assumption that such language is a direct causal product of media exposure.

**Figure 1:**
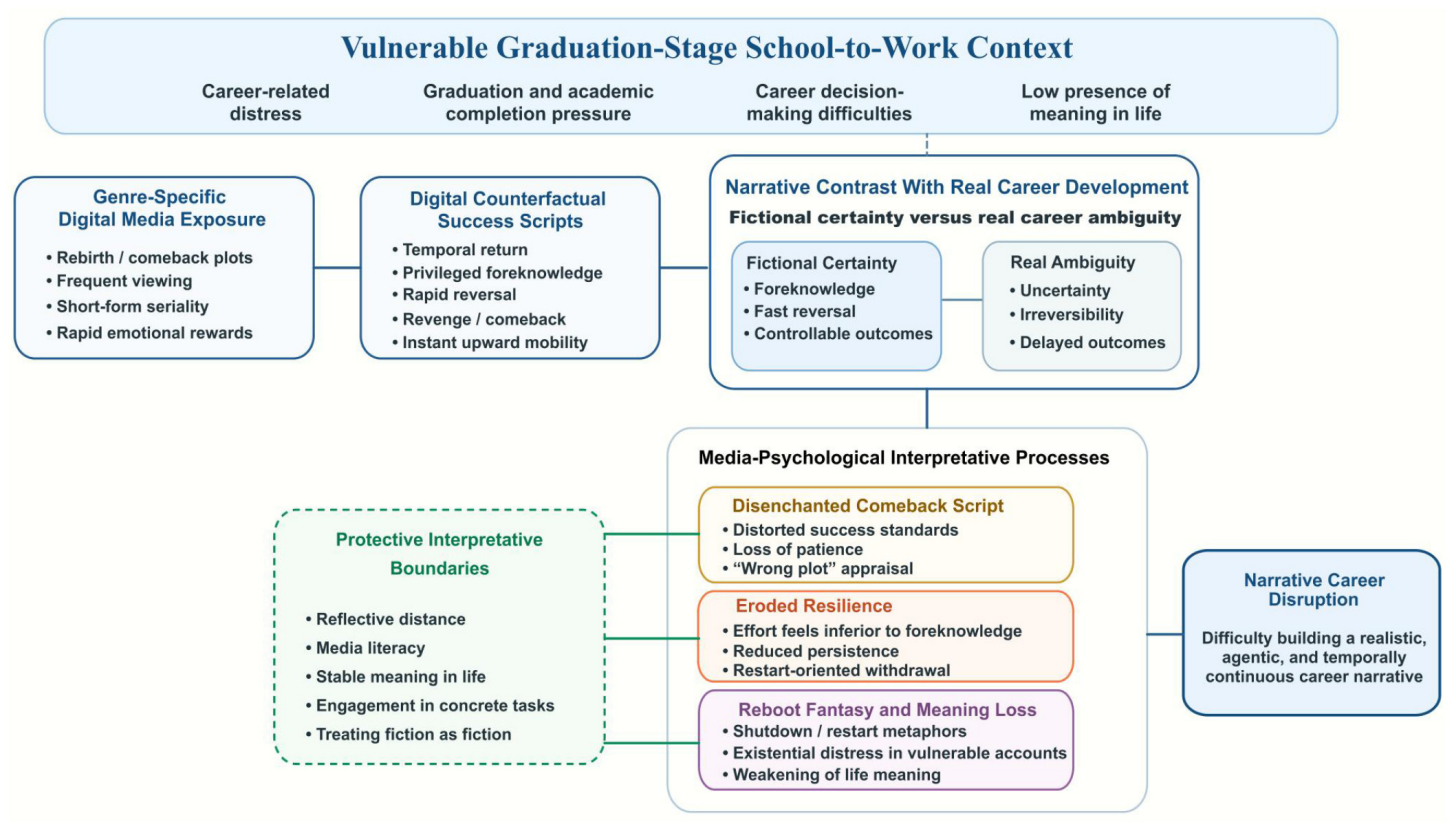
Conceptual framework: rebirth micro-drama immersion and narrative career disruption during the graduation-stage school-to-work transition. Conceptual framework linking rebirth micro-drama immersion, digital counterfactual success scripts, media-psychological interpretative processes, and narrative career disruption among graduation-stage Chinese university students during the school-to-work transition. Arrows indicate interpretative and meaning-making relations reported in participants' accounts rather than temporal order, causal direction, or tested mechanisms. The figure should therefore be read as a sensitizing framework for IPA interpretation, not as a path model.

Taken together, these theoretical perspectives suggest that rebirth micro-drama immersion may shape graduation-stage students' career meaning-making through the contrast between fictional certainty and real-world ambiguity, the narrative devaluation of effort, and reboot-related meaning processes (Green and Brock, 2000; Moyer-Gusé, 2008; Cohen, 2001; Wang and Li, 2024; Savickas and Porfeli, 2012; Rudolph et al., 2017; Savickas et al., 2009; Pordelan et al., 2021, 2018; Gati et al., 1996; Kulcsár et al., 2020; Xu and Tracey, 2015; Luthans and Youssef-Morgan, 2017; Avey et al., 2011; Newman et al., 2014; Luthans et al., 2010; Steger et al., 2006; Martela and Steger, 2016; George and Park, 2016; Heintzelman and King, 2014; Arnett, 2000; Van Orden et al., 2010; Franklin et al., 2017; Xia et al., 2002). These links are treated as sensitizing theoretical pathways for qualitative interpretation, not as hypotheses tested through statistical modeling. Figure 1 presents the conceptual framework guiding the present study.

## 3 Method

### 3.1 Research design

This study adopted interpretative phenomenological analysis (IPA). IPA is designed to explore how individuals make sense of significant experiences in their lives. It is idiographic, interpretative, and phenomenological: it attends closely to individual cases, acknowledges the researcher's interpretative role, and focuses on lived experience (Smith, 1996; Pietkiewicz and Smith, 2014). IPA was chosen because the research questions concern the personal meaning of rebirth micro-drama immersion rather than the prevalence of viewing behavior or causal effects (Smith, 1996; Pietkiewicz and Smith, 2014).

### 3.2 Participants and recruitment

Participants were recruited through university counseling centers, counselor referrals, and social media advertisements. Inclusion criteria were: (a) current enrollment in a graduation-stage university program, defined as either fourth-year undergraduate students preparing for graduation or master's students in the final stage of two- or three-year programs; (b) daily viewing of rebirth or comeback-themed micro-dramas for at least one hour on average during the previous 6 months; and (c) current career-related distress, such as job-search frustration, postgraduate examination failure, repeated changes in career direction, or expressed hopelessness about future development. Students outside the graduation-stage transition were not included. Exclusion criteria were acute psychotic symptoms or immediate suicide risk requiring emergency intervention (Smith, 1996; Pietkiewicz and Smith, 2014; Lakeman and FitzGerald, 2009).

Eighteen participants were included, all of whom were in the graduation-stage school-to-work transition: 12 were fourth-year undergraduate students preparing for graduation, three were in the final semester of two-year master's programs, and three were in the final year of three-year master's programs. Participants were aged between 20 and 24 years (M = 21.94, SD = 1.26). Nine identified as women and nine as men. Participants came from humanities, business, science and engineering, art and design, education, medicine, and foreign language programs. Average daily viewing time was 2.10 hours (SD = 0.61). To address the relatively large size of the IPA sample while preserving idiographic depth, the analysis was organized in two sequential layers rather than as unequal evidentiary groups. Thirteen information-rich cases formed the initial idiographic-development layer and were used to build detailed within-case and cross-case interpretations. Five further cases were then examined through the same transcript-based reading and coding procedures as a supplementary resonance, extension, and negative-case layer. These five cases were not treated as statistically representative, but they were used to check whether the developing themes were supported, extended, or contradicted by additional idiographic material (Smith, 1996; Pietkiewicz and Smith, 2014; Saunders et al., 2018). A summarized sample profile is presented in Table 1; full participant-level profiles, contextual measure scores, and analysis-layer labels are provided in Appendix A (Tables A1 and A2).

**Table 1:** Summary of participant characteristics, viewing behavior, and contextual profiles (*N* = 18).

Variable	Category/stat.	Value	%/SD/range
Sex	Female	9	50.0
	Male	9	50.0
Transition status	Graduating UG senior	12	66.7
	Final-semester 2-year master's	3	16.7
	Final-year 3-year master's	3	16.7
Major type	Humanities	4	22.2
	Business	3	16.7
	STEM	4	22.2
	Art/design	2	11.1
	Education	2	11.1
	Medicine	2	11.1
	Foreign language	1	5.6
Preferred theme	Campus comeback	6	33.3
	Wealth reversal	5	27.8
	Romantic revenge	4	22.2
	Personality reset	3	16.7
Contextual profile	A. Elevated suicide-ideation/career-difficulty context	10	55.6
	B. Career-difficulty prominent context	4	22.2
	C. Lower-risk contextual profile	4	22.2
Analysis layer	Core analysis	13	72.2
	Supplementary check	5	27.8
Age (years)	Mean (SD)	21.94	1.26
	Median (range)	22.00	20–24
Daily viewing time (h)	Mean (SD)	2.10	0.61
	Median (range)	2.15	1.0–3.0

This compact table summarizes the graduation-stage sample for the main text. Graduation-stage status refers to fourth-year undergraduate students preparing for graduation or master's students in the final stage of two- or three-year programs. Contextual profiles were used only as sensitizing descriptors for qualitative interpretation, not as diagnostic categories or statistical comparison groups. Full participant-level profiles and scale scores are provided in Appendix A, Tables A1, A2.

### 3.3 Screening and contextual measures

Three standardized instruments were administered before the interviews: the Self-Rating Idea of Suicide Scale, the Career Decision-Making Difficulties Questionnaire, and the Meaning in Life Questionnaire. These instruments were used to support purposive recruitment, provide case-level contextual information, and assist interpretative triangulation (Gati et al., 1996; Steger et al., 2006; Xia et al., 2002; Yardley, 2000; Tong et al., 2007; Tracy, 2010). They were not used for hypothesis testing, diagnosis, statistical comparison, or causal inference. Given the small and purposive IPA sample, all scale scores were interpreted descriptively and cautiously. The SIOSS score of 12 or above was treated, following the original scale material, as indicating elevated suicide-ideation risk, but it was not used as a clinical diagnosis (Xia et al., 2002). By contrast, the CDDQ and MLQ thresholds used in the contextual profiles were not clinical cut-offs. They were study-specific heuristic descriptors designed to organize the purposive sample's contextual range: CDDQ values were used to indicate relatively prominent career-decision difficulty within this information-rich group, whereas lower MLQ-Presence and higher MLQ-Search values were used to sensitize the analysis to meaning-related distress (Gati et al., 1996; Steger et al., 2006). The SIOSS was used to identify whether participants' accounts involved suicide-related ideation or reboot-related existential distress. The CDDQ was used to contextualize participants' career decision-making difficulties during the school-to-work transition. The MLQ was used to provide background information about participants' presence of meaning and search for meaning. Descriptive profiles of these instruments are reported in the appendices to enhance transparency of the purposive sample rather than to estimate statistical associations, prevalence, or effect sizes (Smith, 1996; Pietkiewicz and Smith, 2014; Yardley, 2000; Tong et al., 2007; Tracy, 2010; Saunders et al., 2018).

### 3.4 Interview procedure

Semi-structured interviews were conducted either face-to-face or through secure video calls. Interviews lasted between 60 and 97 min. The interview guide covered four areas: viewing history and motivation; career imagination and perceived contrast between fiction and reality; persistence, withdrawal, and responses to setbacks; and meaning in life and fantasies of restarting or rebooting life. Participants were encouraged to provide concrete examples rather than general opinions (Smith, 1996; Pietkiewicz and Smith, 2014; Tong et al., 2007; Tracy, 2010).

### 3.5 Data analysis

All interviews were audio-recorded with consent and transcribed verbatim. Data analysis followed the six steps of IPA: repeated reading of each transcript; initial noting of descriptive, linguistic, and conceptual material; development of emergent themes; mapping connections among emergent themes within each case; moving to the next case while bracketing previous interpretations as far as possible; and identifying patterns across cases (Smith, 1996; Pietkiewicz and Smith, 2014). All 18 transcripts were read repeatedly and coded at the case level. The first 13 cases were used as an idiographic-development layer because they provided sufficient depth and variation for building the initial interpretative structure. The subsequent five cases were then analyzed as a resonance, extension, and negative-case layer: they were examined case by case against the emerging structure, and any confirming, extending, or contradictory material was recorded in the audit trail. This staged procedure was used to manage the analytic burden of an 18-participant IPA study while retaining transparency about depth, sequence, and limits; it was not used to claim statistical representativeness, numerical saturation, or prevalence (Smith, 1996; Pietkiewicz and Smith, 2014; Saunders et al., 2018).

### 3.6 Reflexivity, trustworthiness, and ethics

Several strategies were used to enhance trustworthiness and to make the study's adherence to qualitative reporting standards explicit. In line with COREQ and SRQR principles, the research team documented interviewer roles, recruitment procedures, interview settings, consent procedures, reflexive positioning, analytic steps, and evidence linking raw data to themes (Tong et al., 2007; O'Brien et al., 2014). The team maintained reflexive memos throughout the analysis, especially regarding assumptions about digital media, youth culture, career distress, and suicide-related language. Two independent researchers conducted parallel coding on three transcripts, and differences were resolved through discussion. An audit trail was maintained through transcript notes, emergent-theme tables, cross-case theme matrices, and the coding-density summary reported in Appendix E. A summary of the superordinate themes was shared with five participants for member reflection, and their responses were used to clarify wording rather than to override interpretative analysis (Yardley, 2000; Tong et al., 2007; Tracy, 2010; Saunders et al., 2018; O'Brien et al., 2014; Korstjens and Moser, 2018).

The study was reviewed and approved by the Asia-Pacific Think Tank Institute ethics review committee (Approval No. APTTRI-IRB-2026-672014; January 15, 2026). This committee was used as an independent third-party human-subjects review body for a multi-site, non-interventional interview study in which participating universities and counseling contacts assisted with recruitment or referral but did not serve as data controllers and did not receive identifiable research data. The authors' affiliated universities were outside the participant recruitment setting, and the protocol was therefore submitted to this independent review committee before data collection. All participants provided informed consent before participation and were informed of the research purpose, interview procedures, use of screening and contextual measures, data use, privacy protection, voluntary participation, and their right to withdraw at any time without penalty. Interview data were anonymized by removing names, universities, contact information, and other identifying information. Participant identifiers were replaced with codes such as P01–P18, and audio recordings, transcripts, and screening records were stored securely and used only for this study (Lakeman and FitzGerald, 2009).

Because the interviews involved suicide-related metaphors and distress, a risk-management protocol was prepared before data collection. Participants whose screening responses or interview statements indicated immediate suicide risk, operationalized as current intent, plan, inability to commit to short-term safety, or need for emergency intervention, were not included in the research interview or, if already in interview, the interview was to be paused and the participant referred to university counseling or emergency support according to the ethics-approved safety protocol. In the final analytic sample, no interview was terminated for immediate-risk emergency referral. However, 10 participants in the elevated suicide-ideation/career-difficulty contextual profile received enhanced post-interview safety support: the interviewer completed an emotional check-out, provided written and verbal information about university counseling and emergency resources, and confirmed that referral information had been received. Because the study was not a clinical intervention and did not collect treatment-attendance records, the researchers verified delivery and receipt of referral information rather than subsequent counseling uptake. During interviews, researchers avoided suggestive wording when asking about death-related or reboot-related thoughts, allowed participants to pause or withdraw, conducted a brief emotional check-out after the interview, and provided information about university counseling resources to all participants (Lakeman and FitzGerald, 2009).

## 4 Findings

Three superordinate themes were identified: the disenchanted comeback script, eroded resilience, and reboot fantasy with the collapse of meaning. These themes describe a progressive interpretative pattern rather than a deterministic sequence. Participants did not simply imitate media content. Instead, they used rebirth micro-dramas as symbolic resources for interpreting their own career difficulties, emotional pain, and future possibilities.

### 4.1 Theme 1: the disenchanted comeback script—“my life has the wrong plot”

The first theme concerns participants' experience of contrast between fictional success and real career development. Rebirth micro-dramas provided participants with an emotionally powerful script in which success depended on foreknowledge, timing, and decisive reversal. When participants returned to real-life choices, the absence of certainty became more painful.

#### 4.1.1 The fantasy of informational advantage

Many participants described rebirth as attractive because it solved the problem of uncertainty. They did not primarily want supernatural power; they wanted to know which choices would lead to success.

“If I could be reborn, I would return to my first year of university with the next ten years of technology trends in my head. I would choose the right research direction, publish papers, apply for patents, and be recruited before graduation. But in reality, I cannot even tell whether my supervisor's project has a future. Every step feels like it could be the wrong option.” (P08)

#### 4.1.2 Distorted standards of success

Participants also reported that rebirth micro-dramas shaped their standards of success. In these dramas, success is often visible, rapid, and socially recognized: wealth, revenge, romantic validation, status, or public triumph. Several participants began to evaluate their own educational and career progress against this compressed fictional timeline.

“The heroine in the drama creates a brand and becomes rich in three months. I stay up all night to finish a design project and get a B. If I graduate and earn 8,000 yuan a month, is that success? After all these years of study, does that mean I am still a failure?” (P05)

#### 4.1.3 The loss of patience

A recurring concern was the perceived disappearance of patience. Participants described becoming accustomed to rapid emotional rewards in micro-dramas and then finding long-term projects unbearable.

“I even feel impatient watching a 100-episode micro-drama, so I speed it up. In real life, a project may take three years to show results. How am I supposed to face that? I feel as if my patience has been worn away.” (P02)

### 4.2 Theme 2: perceived erosion of resilience—“if I cannot start over, what is the point of trying?”

The second theme concerns participants' interpretation of effort, persistence, and withdrawal. For several participants, rebirth micro-dramas did not simply provide escapism. They changed the emotional meaning of effort. Ordinary effort came to seem inferior, slow, or even foolish compared with the fictional logic of foreknowledge and instant reversal.

“How do the protagonists turn things around? Informational advantage, foreknowledge, a golden finger. Not effort. Effort is worthless compared with rebirth. So what is the meaning of my effort in real life?” (P01)

Graduating undergraduates and final-year master's students described repeated switching of internships, career directions, or job plans. Although such switching can be adaptive during exploration, participants sometimes framed it as an attempt to restart rather than reflectively revise.

“I changed internship directions three times in my final year and changed jobs four times after graduation. Whenever I could not continue, there was a voice in my head saying: it is fine, this account is ruined; just change to another one.” (P06)

Some participants described a rapid slide from concrete problems to global self-negation. Career or academic setbacks became existentially totalized; the failure of a task was interpreted as the failure of the person.

“When my proposal was rejected, I did not think, ‘This topic does not work.' I thought, ‘I am not suitable for being alive.' I know this logic is absurd, but that is how it felt.” (P03)

### 4.3 Theme 3: reboot fantasy and the collapse of meaning—“please let me shut down and restart”

The third theme concerns the existential function of rebirth fantasy. Participants used the language of rebooting, restarting, shutting down, or opening a new file to describe emotional escape. For many, such fantasy was soothing. For some participants in the higher-risk contextual profile, however, it became entangled with suicidal ideation.

“I started looking forward to sleeping. Not because I was tired, but because only after closing my eyes could the reborn version of me be free. The daytime me is a prisoner; the nighttime me is a god.” (P09)

“The more I hate myself during the day, the more I need that fantasy at night. The better the fantasy becomes, the more disgusting I feel in the daytime. It is a dead cycle.” (P15)

The most clinically concerning pattern appeared among several participants whose contextual SIOSS scores and interview accounts suggested more salient suicide-related ideation. They explicitly connected death with the possibility of restarting. These statements do not mean that media exposure alone produced suicidal ideation. Rather, they show how culturally available metaphors may shape the language and imagery through which vulnerable individuals understand death.

“In those dramas, the protagonists are reborn only after they die, right? Sometimes I cannot help thinking: does this terrible life also need to end first before I can open a new file?” (P08)

“I used to think suicide was painful. But rebirth dramas gave me a strange thought—what if death is only a transition? Like a game loading screen: it goes black, and then a new level appears.” (P15)

### 4.4 Negative cases and protective boundaries

Not all participants experienced rebirth micro-dramas as psychologically disruptive. Participants in the lower-risk contextual profile maintained clearer boundaries between fiction and life. These negative cases are theoretically important because they suggest that rebirth micro-drama exposure is not uniformly harmful. Protective factors may include reflective distance, media literacy, stable meaning in life, ongoing engagement in concrete tasks, and the ability to treat fiction as fiction.

“It is just drama. After feeling satisfied for a while, it is over. My thesis still has to be written by myself. The heroine cannot write it for me.” (P07)

“I used to watch them too, but later I found that I felt worse after watching, so I stopped. Now I do experiments and think those plots are ridiculous. Life is not a micro-drama. There are not so many three-minute reversals.” (P17)

## 5 Discussion

This study explored how graduating undergraduate and final-year master's students in China who frequently watched rebirth micro-dramas made sense of career distress and meaning in life. The findings suggest that rebirth micro-dramas may function as powerful digital success scripts for some vulnerable viewers. Participants did not passively absorb these scripts. Instead, they used them to interpret uncertainty, effort, failure, and the desire to start over. Three themes—the disenchanted comeback script, perceived erosion of resilience, and reboot fantasy—together describe a pattern of narrative career disruption. This pattern should be understood as participants' meaning-making around media narratives and career vulnerability, not as evidence that media immersion caused distress.

### 5.1 Narrative career disruption

We propose narrative career disruption as a mid-range interpretative concept to capture the findings. Narrative career disruption refers to a condition in which an individual's ability to construct a realistic, agentic, and temporally continuous career narrative is experienced as weakened when culturally available scripts of certainty, rapid reversal, or escape from irreversibility become more emotionally compelling than gradual real-world development. In the present study, participants compared their own slow and uncertain development with fictional rebirth stories in which success was immediate and guaranteed by foreknowledge. This concept is offered as an interpretative account grounded in the present IPA findings, rather than as a validated psychological construct, causal mechanism, or claim that rebirth micro-dramas independently produce career distress.

This concept extends career construction theory by emphasizing not only deficits in adaptability but also competition among narratives. Students may have the cognitive ability to explore careers, yet still experience their personal narratives as pressured by more emotionally rewarding cultural scripts. When the fictional script feels more coherent, more satisfying, and more decisive than the real-life story, career construction may become psychologically harder for vulnerable viewers. This formulation leaves open the reverse and reciprocal possibilities that students with weakened resilience or career despair may be especially likely to seek out rebirth narratives for compensation. The contextual profiles used to support this qualitative interpretation are summarized in Table 2.

**Table 2:** Contextual profiles used to support qualitative interpretation.

Contextual profile	*n*	SIOSS M (SD)	CDDQ M (SD)	MLQ-presence M (SD)	Theme 1	Theme 2	Theme 3	Core interpretative feature
A. Elevated suicide-ideation/career-difficulty context	10	19.10 (2.60)	126.30 (8.07)	9.70 (2.31)	Deep	Deep	Very dense	Full disruption spectrum
B. Career-difficulty prominent context	4	12.25 (1.71)	133.00 (12.36)	13.00 (2.94)	Moderate	Deep	Shallow	Perceived resilience weakening dominant
C. Lower-risk contextual profile	4	7.25 (1.71)	106.25 (9.60)	19.75 (1.71)	Shallow	Shallow	None	Reality boundary maintained
Full sample	18	14.94 (5.51)	123.33 (13.17)	12.67 (4.68)	—	—	—	—

Contextual profiles were used as sensitizing descriptors to support qualitative interpretation. They were not diagnostic categories, statistical comparison groups, or indicators of prevalence. Theme engagement was interpreted qualitatively on the basis of the number, density, and interpretative salience of emergent themes in the IPA audit trail; it should not be read as an effect size.

### 5.2 Psychological capital and narrative devaluation of effort

The findings also contribute to psychological capital theory. Participants' accounts suggest that psychological resources may be experienced as weakened not only after repeated objective failure but also through the narrative devaluation of effort. In traditional models, self-efficacy may decline because individuals try and fail. In the present study, some participants questioned effort before sustained effort could occur because the media narratives they consumed made ordinary pathways appear weak compared with rebirth-enabled success. This wording should be read as interpretative: the interviews cannot determine whether micro-drama viewing weakened resilience, whether pre-existing vulnerability increased attraction to such narratives, or whether both processes reinforced each other.

This does not imply that psychological capital theory is insufficient. Rather, it suggests that psychological capital is embedded in narrative environments. Hope requires pathways that feel meaningful; resilience requires setbacks that feel survivable; optimism requires a future that feels open but not impossible. Rebirth micro-dramas may become psychologically risky for certain viewers when, in their own interpretation, realistic pathways appear too slow to matter.

### 5.3 Reboot fantasy and suicide risk language

A clinically significant finding is that some participants used reboot language to describe death or suicidal thoughts. This does not establish a causal relationship between rebirth micro-drama viewing and suicidal ideation. However, it highlights a potential cultural marker of risk. In suicide assessment, practitioners often attend to hopelessness, burdensomeness, planning, and access to means. The present findings suggest that clinicians and university counselors should also attend to metaphors such as shutting down, restarting, opening a new file, or ending this timeline, especially when these metaphors are emotionally linked with death. Such metaphors should be interpreted within a full clinical assessment rather than treated as diagnostic indicators on their own, and their presence should prompt careful inquiry rather than automatic pathologization of media consumption.

### 5.4 Interdisciplinary contribution

The study is primarily situated in vocational and applied psychology, but it has interdisciplinary implications. For cyberpsychology, it shows that the psychological significance of digital media depends not only on usage time but also on narrative structure, platform delivery, interpretative context, and personal vulnerability. For positive organizational behavior, it suggests that psychological capital should be examined before formal employment, during the school-to-work transition, and in relation to digital cultural scripts. For human resource management, the findings point to the importance of expectation management among new entrants to the workforce.

### 5.5 Practical implications

The findings suggest several practical implications. First, university career counseling may benefit from narrative assessment. Counselors can ask students not only what occupations they prefer, but also what stories about success, failure, time, and effort they have absorbed from digital media. Second, media literacy programs should be integrated with career development education. Such programs should avoid moral panic or simple prohibition and instead help students distinguish fictional compression, algorithmic reward, and real developmental time. Third, suicide risk assessment should include attention to reboot metaphors when working with students who consume rebirth-themed content, while still treating such metaphors as prompts for careful inquiry rather than as standalone indicators of risk. Fourth, employers and human resource professionals may consider narrative expectation management during onboarding.

### 5.6 Limitations and future research

This study has several limitations. First, it used a purposive and relatively small sample, consistent with IPA but not suitable for statistical generalization. At the same time, 18 participants is relatively large for conventional IPA; we therefore used a staged idiographic-development and resonance/negative-case procedure to preserve depth while increasing transparency about analytic scope. The findings should be interpreted as an in-depth account of a specific group: frequent rebirth micro-drama viewers experiencing career-related distress. Second, the standardized instruments were used for screening, case-level contextualization, and interpretative triangulation, not for diagnosis, causal inference, or statistical comparison. No p-values, effect sizes, inferential tests, or norm-based claims are made from the screening data; score summaries and contextual profiles are used only as descriptive background for interpreting the qualitative cases. Third, although the contextual profiles helped describe the sample's range, they should not be treated as natural kinds or fixed boundaries among participants' experiences. Fourth, the cross-sectional interview design cannot determine directionality. It remains possible that students with already weakened resilience, lower meaning in life, or greater career despair were more likely to seek out rebirth micro-dramas for psychological compensation. Future studies should use larger samples and longitudinal designs to test whether narrative internalization predicts changes in career adaptability, psychological capital, and meaning in life. Experimental or diary methods could examine short-term emotional and cognitive responses to different types of micro-drama narratives. Fifth, the study focused on Chinese university students; comparative studies could examine similar narratives in Korean webtoons, Japanese light novels, Western time-loop fiction, or gaming cultures.

## 6 Conclusion

This study examined how graduating undergraduate and final-year master's students in China who frequently watched rebirth micro-dramas interpreted their career difficulties and meaning in life. The findings suggest that rebirth micro-dramas can become more than entertainment for some vulnerable viewers. They may provide a powerful script of certainty, reversal, and instant success that contrasts sharply with the slow, ambiguous, and irreversible nature of real career development. Through this contrast, participants described disenchanted career imagination, perceived erosion of resilience, and, in some cases, reboot fantasies connected with existential distress and suicidal ideation. These findings should be understood as an interpretative account of a vulnerable purposive sample, not as evidence that rebirth micro-dramas are uniformly harmful or causally sufficient to produce distress.

The study contributes to vocational psychology by proposing narrative career disruption as a way to understand how digital cultural scripts may become involved in career meaning-making. It contributes to psychological capital theory by identifying a narrative pathway through which effort, hope, and resilience may be interpreted as devalued by some vulnerable viewers. Finally, it highlights the need for career counseling, media literacy, and mental health support that take young adults' digital narrative environments seriously.

## Data Availability

The raw data supporting the conclusions of this article will be made available by the authors, without undue reservation.

## References

[B1] Arnett J. J. (2000). Emerging adulthood: a theory of development from the late teens through the twenties. Am. Psychol. 55, 469–480. doi: 10.1037/0003-066X.55.5.469 10842426

[B2] Avey J. B. Reichard R. J. Luthans F. Mhatre K. H. (2011). Meta-analysis of the impact of positive psychological capital on employee attitudes, behaviors, and performance. Hum. Resour. Dev. Q. 22, 127–152. doi: 10.1002/hrdq.20070

[B3] Chen Y. Li M. Guo F. Wang X. (2023). The effect of short-form video addiction on users' attention. Behav. Inf. Technol. 42, 2893–2910. doi: 10.1080/0144929X.2022.2151512

[B4] Cohen J. (2001). Defining identification: a theoretical look at the identification of audiences with media characters. Mass Commun. Soc. 4, 245–264. doi: 10.1207/S15327825MCS0403_01

[B5] Coyne S. M. Rogers A. A. Zurcher J. D. Stockdale L. Booth M. (2020). Does time spent using social media impact mental health?: an eight year longitudinal study. Comput. Human Behav. 104:106160. doi: 10.1016/j.chb.2019.106160

[B6] Franklin JC. Ribeiro JD. Fox KR. Bentley KH. Kleiman EM. Huang X. . (2017). Risk factors for suicidal thoughts and behaviors: a meta-analysis of 50 years of research. Psychol. Bull. 143, 187–232. doi: 10.1037/bul0000084 27841450

[B7] Gati I. Krausz M. Osipow S. H. (1996). A taxonomy of difficulties in career decision making. J. Couns. Psychol. 43, 510–526. doi: 10.1037/0022-0167.43.4.510

[B8] George L. S. Park C. L. (2016). Meaning in life as comprehension, purpose, and mattering: toward integration and new research questions. Rev. Gen. Psychol. 20, 205–220. doi: 10.1037/gpr0000077

[B9] Green M. C. Brock T. C. (2000). The role of transportation in the persuasiveness of public narratives. J. Pers. Soc. Psychol. 79, 701–721. doi: 10.1037/0022-3514.79.5.701 11079236

[B10] Heintzelman S. J. King L. A. (2014). Life is pretty meaningful. Am. Psychol. 69, 561–574. doi: 10.1037/a0035049 24490907

[B11] KorstjensI. MoserA. (2018). Series: practical guidance to qualitative research. Part 4: trustworthiness and publishing. Eur. J. Gen. Pract. 24, 120–124. doi: 10.1080/13814788.2017.137509229202616 PMC8816392

[B12] Kulcsár V. Dobrean A. Gati I. (2020). Challenges and difficulties in career decision making: their causes, and their effects on the process and the decision. J. Vocat. Behav. 116:103346. doi: 10.1016/j.jvb.2019.103346

[B13] Lakeman R. FitzGerald M. (2009). The ethics of suicide research: the views of ethics committee members. Crisis 30, 13–19. doi: 10.1027/0227-5910.30.1.13 19261563

[B14] LiuY. NiX. NiuG. (2021). Perceived stress and short-form video application addiction: a moderated mediation model. Front. Psychol. 12:747656. doi: 10.3389/fpsyg.2021.74765635002843 PMC8733249

[B15] Luthans F. Avey J. B. Avolio B. J. Peterson S. J. (2010). The development and resulting performance impact of positive psychological capital. Hum. Resour. Dev. Q. 21, 41–67. doi: 10.1002/hrdq.20034

[B16] Luthans F. Youssef-Morgan C. M. (2017). Psychological capital: an evidence-based positive approach. Annu. Rev. Organ. Psychol. Organ. Behav. 4, 339–366. doi: 10.1146/annurev-orgpsych-032516-113324

[B17] MaoM. LiaoF. (2025). Undergraduates short form video addiction and learning burnout association involving anxiety symptoms and coping styles moderation. Sci. Rep. 15:24191. doi: 10.1038/s41598-025-09656-x40624123 PMC12234783

[B18] Martela F. Steger M. F. (2016). The three meanings of meaning in life: distinguishing coherence, purpose, and significance. J. Posit. Psychol. 11, 531–545. doi: 10.1080/17439760.2015.1137623

[B19] MontagC. YangH. ElhaiJ. D. (2021). On the psychology of TikTok use: a first glimpse from empirical findings. Front. Public Health 9:641673. doi: 10.3389/fpubh.2021.64167333816425 PMC8010681

[B20] Moyer-Gusé E. (2008). Toward a theory of entertainment persuasion: explaining the persuasive effects of entertainment-education messages. Commun. Theory 18, 407–425. doi: 10.1111/j.1468-2885.2008.00328.x

[B21] Newman A. Ucbasaran D. Zhu F. Hirst G. (2014). Psychological capital: a review and synthesis. J. Organ. Behav. 35, S120–S138. doi: 10.1002/job.1916

[B22] O'Brien BC. Harris IB. Beckman TJ. Reed DA. Cook DA. (2014). Standards for reporting qualitative research: a synthesis of recommendations. Acad. Med. 89, 1245–1251. doi: 10.1097/ACM.0000000000000388 24979285

[B23] OdgersC. L. JensenM. R. (2020). Annual research review: adolescent mental health in the digital age: facts, fears, and future directions. J. Child Psychol. Psychiatry 61, 336–348. doi: 10.1111/jcpp.1319031951670 PMC8221420

[B24] Pietkiewicz I. Smith J. A. (2014). A practical guide to using Interpretative Phenomenological Analysis in qualitative research psychology. Czas. Psychol. / Psychol. J. 20, 7–14. doi: 10.14691/CPPJ.20.1.7

[B25] Pordelan N. Hosseinian S. Baei Lashaki A. (2021). Digital storytelling: a tool for life design career intervention. Educ. Inf. Technol. 26, 3445–3457. doi: 10.1007/s10639-020-10403-0

[B26] Pordelan N. Sadeghi A. Abedi M. R. Kaedi M. (2018). How online career counseling changes career development: a life design paradigm. Educ. Inf. Technol. 23, 2655–2672. doi: 10.1007/s10639-018-9735-1

[B27] QinY. OmarB. MusettiA. (2022). The addiction behavior of short-form video app TikTok: the information quality and system quality perspective. Front. Psychol. 13:932805. doi: 10.3389/fpsyg.2022.93280536148123 PMC9486470

[B28] Rudolph C. W. Lavigne K. N. Zacher H. (2017). Career adaptability: a meta-analysis of relationships with measures of adaptivity, adapting responses, and adaptation results. J. Vocat. Behav. 98, 17–34. doi: 10.1016/j.jvb.2016.09.002

[B29] SaundersB. SimJ. KingstoneT. BakerS. WaterfieldJ. BartlamB. . (2018). Saturation in qualitative research: exploring its conceptualization and operationalization. Qual. Quant. 52, 1893–1907. doi: 10.1007/s11135-017-0574-829937585 PMC5993836

[B30] Savickas M. L. Nota L. Rossier J. Dauwalder J-. P. Eduarda Duarte M. Guichard J. . (2009). Life designing: a paradigm for career construction in the 21st century. J. Vocat. Behav. 75, 239–250. doi: 10.1016/j.jvb.2009.04.004

[B31] Savickas M. L. Porfeli E. J. (2012). Career Adapt-Abilities Scale: construction, reliability, and measurement equivalence across 13 countries. J. Vocat. Behav. 80, 661–673. doi: 10.1016/j.jvb.2012.01.011

[B32] Smith J. A. (1996). Beyond the divide between cognition and discourse: using interpretative phenomenological analysis in health psychology. Psychol. Health 11, 261–271. doi: 10.1080/08870449608400256

[B33] Steger M. F. Frazier P. Oishi S. Kaler M. (2006). The Meaning in Life Questionnaire: assessing the presence of and search for meaning in life. J. Couns. Psychol. 53, 80–93. doi: 10.1037/0022-0167.53.1.80

[B34] Tong A. Sainsbury P. Craig J. (2007). Consolidated criteria for reporting qualitative research (COREQ): a 32-item checklist for interviews and focus groups. Int. J. Qual. Health Care 19, 349–357. doi: 10.1093/intqhc/mzm042 17872937

[B35] Tracy S. J. (2010). Qualitative quality: eight “big-tent” criteria for excellent qualitative research. Qual. Inq. 16, 837–851. doi: 10.1177/1077800410383121

[B36] Valkenburg P. M. Meier A. Beyens I. (2022). Social media use and its impact on adolescent mental health: an umbrella review of the evidence. Curr. Opin. Psychol. 44, 58–68. doi: 10.1016/j.copsyc.2021.08.017 34563980

[B37] Van OrdenKA. WitteTK. CukrowiczKC. BraithwaiteSR. SelbyEA. JoinerTE. (2010). The interpersonal theory of suicide. Psychol. Rev. 117, 575–600. doi: 10.1037/a001869720438238 PMC3130348

[B38] Verduyn P. Gugushvili N. Massar K. Täht K. Kross E. (2020). Social comparison on social networking sites. Curr. Opin. Psychol. 36, 32–37. doi: 10.1016/j.copsyc.2020.04.002 32387840

[B39] WangD. LiY. (2024). Career construction theory: tools, interventions, and future trends. Front. Psychol. 15:1381233. doi: 10.3389/fpsyg.2024.138123338646130 PMC11026660

[B40] Xia C. Y. Wang D. B. Wu S. Q. Ye J. H. (2002). Preliminary development of self-rating scale for suicidal ideation. J. Clin. Psychiatry 12, 100–102. doi: 10.3969/j.issn.1005-3220.2002.02.030

[B41] XieJ. XuX. ZhangY. TanY. WuD. ShiM. . (2023). The effect of short-form video addiction on undergraduates' academic procrastination: a moderated mediation model. Front. Psychol. 14:1298361. doi: 10.3389/fpsyg.2023.129836138162977 PMC10756502

[B42] Xu H. Tracey T. J. G. (2015). Career Decision Ambiguity Tolerance Scale: construction and initial validations. J. Vocat. Behav. 88, 1–9. doi: 10.1016/j.jvb.2015.01.006

[B43] Yardley L. (2000). Dilemmas in qualitative health research. Psychol. Health 15, 215–228. doi: 10.1080/08870440008400302

[B44] Zhang X. Wu Y. Liu S. (2019). Exploring short-form video application addiction: socio-technical and attachment perspectives. Telemat. Inform. 42:101243. doi: 10.1016/j.tele.2019.101243

