# Supplementary material for: “Shut down and restart”: immersion in rebirth micro-dramas and narrative career disruption among Chinese university students—an interpretative phenomenological analysis
Source: Front Psychol. 2026 Jul 22;17:1887188. doi: 10.3389/fpsyg.2026.1887188 (PMC13439647; doi:10.3389/fpsyg.2026.1887188)
Supplement: Supplementary file 1 [file Data_Sheet_1.docx]

**Appendix**

**Appendix A. Full Participant Profiles, Contextual Measure Scores, and Analysis Layer**

**Table A1. Participant Profiles and Analysis Layer (N = 18)**

| **ID** | **Sex** | **Age** | **Status** | **Major** | **Daily h** | **Theme** | **Profile** | **Layer** |
| --- | --- | --- | --- | --- | --- | --- | --- | --- |
| P01 | F | 22 | UG senior | Humanities | 2.5 | Campus | A | Core |
| P02 | M | 21 | UG senior | Business | 1.8 | Wealth | B | Core |
| P03 | F | 24 | MA-2 final | Humanities | 3.0 | Romantic | A | Core |
| P04 | M | 22 | UG senior | STEM | 1.5 | Campus | C | Core |
| P05 | F | 20 | UG senior | Art/design | 2.2 | Personality | A | Core |
| P06 | M | 23 | MA-2 final | Business | 2.0 | Wealth | B | Core |
| P07 | F | 22 | UG senior | Education | 1.2 | Campus | C | Core |
| P08 | M | 21 | UG senior | STEM | 2.8 | Wealth | A | Core |
| P09 | F | 22 | UG senior | Medicine | 2.1 | Personality | A | Core |
| P10 | M | 23 | MA-3 final | STEM | 1.0 | Campus | C | Core |
| P11 | F | 20 | UG senior | Humanities | 2.6 | Romantic | A | Core |
| P12 | M | 23 | MA-2 final | Business | 1.9 | Wealth | B | Core |
| P13 | F | 21 | UG senior | Foreign lang. | 2.4 | Romantic | A | Core |
| P14 | M | 22 | UG senior | STEM | 1.6 | Campus | B | Supp. |
| P15 | F | 24 | MA-3 final | Education | 2.9 | Personality | A | Supp. |
| P16 | M | 20 | UG senior | Art/design | 2.3 | Wealth | A | Supp. |
| P17 | F | 22 | UG senior | Medicine | 1.3 | Campus | C | Supp. |
| P18 | M | 23 | MA-3 final | Humanities | 2.7 | Romantic | A | Supp. |

**Table A2. Contextual Measure Scores by Participant (N = 18)**

| **ID** | **SIOSS** | **CDDQ** | **Lack prep.** | **Lack info.** | **Inconsistent info.** | **MLQ-Presence** | **MLQ-Search** |
| --- | --- | --- | --- | --- | --- | --- | --- |
| P01 | 18 | 128 | 45 | 52 | 31 | 12 | 27 |
| P02 | 12 | 135 | 48 | 56 | 31 | 15 | 24 |
| P03 | 22 | 110 | 38 | 45 | 27 | 8 | 30 |
| P04 | 9 | 120 | 42 | 50 | 28 | 18 | 22 |
| P05 | 20 | 132 | 47 | 53 | 32 | 10 | 28 |
| P06 | 14 | 140 | 50 | 58 | 32 | 11 | 25 |
| P07 | 5 | 98 | 34 | 40 | 24 | 22 | 18 |
| P08 | 19 | 125 | 44 | 51 | 30 | 9 | 31 |
| P09 | 16 | 118 | 40 | 49 | 29 | 13 | 26 |
| P10 | 7 | 105 | 36 | 43 | 26 | 20 | 20 |
| P11 | 21 | 130 | 46 | 54 | 30 | 7 | 33 |
| P12 | 13 | 142 | 52 | 57 | 33 | 10 | 23 |
| P13 | 17 | 122 | 43 | 50 | 29 | 11 | 29 |
| P14 | 10 | 115 | 40 | 48 | 27 | 16 | 21 |
| P15 | 23 | 127 | 45 | 51 | 31 | 6 | 32 |
| P16 | 15 | 138 | 49 | 56 | 33 | 12 | 27 |
| P17 | 8 | 102 | 35 | 42 | 25 | 19 | 19 |
| P18 | 20 | 133 | 47 | 54 | 32 | 9 | 30 |

Note. UG senior = fourth-year undergraduate student preparing for graduation; MA-2 final = final semester of a two-year master's program; MA-3 final = final year of a three-year master's program; Foreign lang. = foreign language; Supp. = supplementary check. SIOSS = Self-Rating Idea of Suicide Scale; CDDQ = Career Decision-Making Difficulties Questionnaire; MLQ = Meaning in Life Questionnaire. Contextual profile A = elevated suicide-ideation and career-decision difficulty context; B = career-decision difficulty prominent context; C = lower-risk contextual profile. Core analysis refers to the first 13 cases analyzed idiographically in detail; supplementary check refers to the five cases examined after the idiographic analysis to assess resonance, extension, and possible contradiction of the developed themes. Values in Tables A1 and A2 are used as participant-level background for qualitative interpretation and descriptive transparency. The profile labels and thresholds are used only as a heuristic for describing the sample's contextual range; they do not imply natural kinds or diagnostic boundaries.

**Appendix B. Screening and Contextual Measures**

The Self-Rating Idea of Suicide Scale (SIOSS) contains 26 dichotomously scored items and includes hopelessness, optimism, sleep, and disguise-related components. The original material treated a total score of 12 or above as indicating elevated suicide-ideation risk. In this study, SIOSS was used only as a screening and contextual risk-sensitizing tool; it was not used to diagnose suicidality or to rank participants clinically. Internal consistency in the current sample was reported as Cronbach's alpha = .87.

The Career Decision-Making Difficulties Questionnaire (CDDQ) contains 34 items rated on a 9-point scale. The original material used three dimensions: lack of preparation, lack of information, and inconsistent information. No universal clinical cut-off was applied in this study; CDDQ thresholds in Table C2 were study-specific heuristic descriptors of prominent career-decision difficulty within this purposive sample. Internal consistency in the current sample was reported as Cronbach's alpha = .92.

The Meaning in Life Questionnaire (MLQ) contains 10 items rated on a 7-point scale. The Presence and Search subscales each contain five items. MLQ thresholds in Table C2 were used only to sensitize the qualitative analysis to low presence of meaning and high search for meaning; they were not treated as clinical thresholds. Internal consistency in the current sample was reported as alpha = .91 for Presence and alpha = .87 for Search.

**Appendix C. Descriptive Statistics and Contextual Profiles**

**Table C1. Descriptive Statistics of Measures and Viewing Behavior (N = 18)**

| **Variable** | **M** | **SD** | **Mdn** | **Min** | **Max** | **Skewness** | **Kurtosis** |
| --- | --- | --- | --- | --- | --- | --- | --- |
| SIOSS total | 14.94 | 5.51 | 15.50 | 5 | 23 | -0.29 | -1.10 |
| CDDQ total | 123.33 | 13.17 | 126.00 | 98 | 142 | -0.50 | -0.69 |
| CDDQ-Lack of preparation | 43.39 | 5.29 | 44.50 | 34 | 52 | -0.33 | -0.83 |
| CDDQ-Lack of information | 50.50 | 5.26 | 51.00 | 40 | 58 | -0.57 | -0.48 |
| CDDQ-Inconsistent information | 29.44 | 2.75 | 30.00 | 24 | 33 | -0.54 | -0.75 |
| MLQ-Presence | 12.67 | 4.68 | 11.50 | 6 | 22 | 0.61 | -0.63 |
| MLQ-Search | 25.83 | 4.60 | 26.50 | 18 | 33 | -0.18 | -1.10 |
| Daily viewing time (h) | 2.10 | 0.61 | 2.15 | 1.0 | 3.0 | -0.27 | -0.98 |

**Table C2. Contextual Profiles and Meaning-in-Life Profile**

| **Contextual profile** | **Heuristic criterion** | **n** | **%** |
| --- | --- | --- | --- |
| A. Elevated suicide-ideation / career-difficulty context | SIOSS >= 15, CDDQ >= 110, MLQ-Presence < 15 (heuristic combination; SIOSS >=12 indicates elevated risk, while CDDQ/MLQ values are study-specific descriptors) | 10 | 55.6 |
| B. Career-difficulty prominent context | After A/C assignment: SIOSS 10-14 and CDDQ >= 115 (study-specific heuristic descriptor of prominent career-decision difficulty) | 4 | 22.2 |
| C. Lower-risk contextual profile | SIOSS < 10, MLQ-Presence > 15 (study-specific lower-risk descriptor, not a clinical category) | 4 | 22.2 |
| Meaning-crisis profile | MLQ-Presence < 18, MLQ-Search > 22 (study-specific meaning-distress descriptor, not a diagnostic threshold) | 13 | 72.2 |

Note. Contextual profiles were used as sensitizing sampling and interpretative descriptors rather than diagnostic categories, statistical comparison groups, or prevalence estimates. A = elevated suicide-ideation and career-decision difficulty context; B = career-decision difficulty prominent context; C = lower-risk contextual profile. Assignment was treated as mutually exclusive only for the purpose of contextualizing the qualitative themes. Except for the original SIOSS risk reference point, the profile thresholds were study-specific heuristic descriptors constructed to summarize the purposive sample's contextual range; they should not be interpreted as standard clinical cut-offs, natural kinds, or diagnostic boundaries.

**Appendix D. Semi-Structured Interview Guide**

1. How did you first begin watching rebirth micro-dramas? Which drama first made you feel highly engaged?

2. Can you describe how you feel when you are immersed in such dramas?

3. At what moments in daily life do you most want to escape into such storylines?

4. What common characteristics do your preferred protagonists have? Do you see any similarity between yourself and them?

5. Have you ever imagined turning your own life around as precisely as a protagonist does?

6. When you receive a rejection, fail an interview, or experience an academic setback, do any scenes from these dramas come to mind?

7. What does career success mean to you? Where do you think this standard comes from?

8. Compared with the protagonist’s comeback path, how do you view your own career future?

9. Can you describe a concrete example of deciding whether to persist or give up?

10. When you want to give up, what thought eventually becomes dominant?

11. Do you feel that after watching many rebirth dramas, you have less patience for things that require a long time to show results?

12. If you used a game metaphor for your life, which level would you be in now? Have you thought about changing account or starting over?

13. Have you ever felt that it would be better if you could completely restart life?

14. Is this feeling related to the rebirth plots you have watched?

15. When you imagine rebooting life, does it help you temporarily escape pain, or does it make real life feel heavier?

16. What helps you continue in a reality without a reset button?

**Appendix E. Emergent Theme Counts and Coding Density as an Audit-Trail Aid**

Table E1. Emergent Theme Counts by Participant and Analysis Layer

| **ID** | **Contextual profile** | **Theme 1** | **Theme 2** | **Theme 3** | **Total emergent themes** | **Coding density** | **Analysis layer** |
| --- | --- | --- | --- | --- | --- | --- | --- |
| P01 | A | 6 | 7 | 5 | 18 | 42 | Core |
| P02 | B | 4 | 8 | 2 | 14 | 38 | Core |
| P03 | A | 7 | 6 | 9 | 22 | 55 | Core |
| P04 | C | 2 | 2 | 0 | 4 | 21 | Core |
| P05 | A | 5 | 8 | 7 | 20 | 48 | Core |
| P06 | B | 3 | 9 | 1 | 13 | 40 | Core |
| P07 | C | 1 | 1 | 0 | 2 | 18 | Core |
| P08 | A | 6 | 7 | 8 | 21 | 52 | Core |
| P09 | A | 5 | 5 | 6 | 16 | 44 | Core |
| P10 | C | 2 | 2 | 0 | 4 | 20 | Core |
| P11 | A | 6 | 7 | 10 | 23 | 58 | Core |
| P12 | B | 3 | 8 | 2 | 13 | 36 | Core |
| P13 | A | 5 | 6 | 7 | 18 | 45 | Core |
| P14 | B | 4 | 7 | 1 | 12 | 35 | Supplementary |
| P15 | A | 7 | 8 | 9 | 24 | 60 | Supplementary |
| P16 | A | 5 | 6 | 6 | 17 | 43 | Supplementary |
| P17 | C | 1 | 2 | 0 | 3 | 19 | Supplementary |
| P18 | A | 6 | 7 | 7 | 20 | 50 | Supplementary |

Note. Emergent theme count refers to the number of independent emergent themes assigned to a participant under each superordinate theme. Coding density refers to the number of transcript passages assigned at least one code. These counts are provided only as an audit-trail aid for qualitative transparency. They should not be interpreted as quantitative indicators of effect size, prevalence, or statistical group differences. For supplementary cases, counts reflect focused theme-resonance coding rather than full idiographic case presentation. The table has been checked to ensure that all cells contain readable numeric or categorical audit-trail information rather than formatting artefacts.
